# Parrots in the wild in Polish cities

**DOI:** 10.1371/journal.pone.0304484

**Published:** 2024-06-20

**Authors:** Agnieszka Ważna, Mateusz Ciepliński, Weronika Ratajczak, Jacek Bojarski, Jan Cichocki

**Affiliations:** 1 Department of Zoology, Institute of Biological Science, University of Zielona Góra, Prof. Z. Szafrana 1, Zielona Góra, Poland; 2 Faculty of Biological Sciences, Student Scientific Club of Biologists, University of Zielona Góra, Prof. Z. Szafrana 1, Zielona Góra, Poland; 3 Institute of Mathematics, Center for Applied Mathematics and Computer Science, University of Zielona Góra, Prof. Z. Szafrana 4a, Zielona Góra, Poland; Auburn University, UNITED STATES

## Abstract

Amateur breeding of parrots as pets has contributed to many species being found in areas where they never occurred in the wild, particularly in warmer regions, but also in the temperate climates of North America and Europe. Climate change is likely to exacerbate this process. We hypothesised that parrots occurred in the wild in the cities of Poland, especially the rose-ringed parakeet, as there are reports of breeding sites in the literature. Using information on lost, found and sighted parrots posted on social media, we have analysed the extent of parrot emergence in Poland. In a period of less than two years (from October 2018, through 2019 and from June to the end of December 2021), 2,675 parrot specimens of 49 species were found in the wild. The most frequently observed species were cockatiel *Nymphicus hollandicus* (N = 962), budgerigar *Melopsittacus undulatus* (N = 884) and rose-ringed parakeet *Psittacula krameri* (N = 182). Parrots were most frequently observed in urban areas, in regions characterized by a higher population size, a higher income and a higher proportion of people with a university degree. Our study shows that the occurrence of parrots, especially rose-ringed parakeets in the wild, needs to be monitored as new breeding sites may appear.

## Introduction

Psittacidae parrots are among the most popular birds chosen by amateur breeders as a pets [1–3]. As a result, they are traded worldwide and transported to regions where they are not found in the wild. They are then intentionally or accidentally released into the wild, where they often acclimate to local environmental conditions [4–14]. Currently, an estimated 120 parrot species have escaped from captivity into the wild and 71 of them have naturalized [15].

Most cases of naturalization are recorded in the cities where many parrot species successfully establish isolated breeding populations [15–18]. There is no consensus on how many parrot species are naturalized in Europe. The most recent studies list 23 parrot species [16]. 11 parrot species are recorded in the EASIN-European Alien Species Information Network [19]. Two species: the monk parakeet *Myiopsitta monachus* and the rose-ringed parakeet *Psittacula krameri* have "high impact" status, which describes their environmental and socio-economic impact, e.g. damage to agriculture and infrastructure, competition with native bird species for food and breeding sites [16].

Parrots are mainly found in the southern and western parts of the continent [16,20–22]. Most parrot species have been naturalized in Spain [15]. The most numerous introduced parrot species in Europe is the rose-ringed parakeet, forming 95 populations of 85,000 individuals [23] or even more than 90,000 individuals [16]. In southern Europe, parrots are also found outside urban areas and cause measurable economic losses in agriculture [24–27] and damage to electricity grids [15].

In Central Europe, the number of breeding non-native bird species is lower than in the west of the continent, mainly due to the temperate climate, and varies between 5–8 species [28]. In Poland, there are officially six bird species that make up the breeding population: Canada goose *Brandta canadensis*, pheasant *Phasianus colchicus*, Mandarin duck *Aix galericulata*, Egyptian goose *Alopochen aegyptiacus*, ruddy shelduck *Tadorna ferruginea*, and rock dove *Columba livia*. The number of breeding non-native bird species has increased in recent years [29,30]. According to Polish ornithological literature, parrots are found accidentally in the wild [31–42]. The opinion-forming Alien Species in Poland database maintained by the Institute of Nature Conservation PAS, contains 7 parrot species. For the budgerigar *Melopsittacus undulatus* and cockatiel *Nymphicus hollandicus*, no localities were given, and for the crimson rosella *Platycercus elegans*, the regent parrot *Polytelis anthopeplus*, the Lord Derby’s parakeet *Psittacula derbiana* and the Alexandrine parakeet *Psittacula eupatria* the inclusion in the database was based on a single observation [30]. The listed parrot species have the status of species that occur only sporadically in Poland and do not breed. The rose-ringed parakeet also has the status of a non-breeding species in Poland in this database. The lack of breeding records for this species in the world literature, although it was recorded in 2018 in Nysa, a small town in the southwest of the country, could be attributed to its non-breeding status. A flock of 5–7 individuals was observed there throughout the year, and 8 individuals were recorded at one site [41,43]. These publications undoubtedly confirm the first breeding of the rose-ringed parakeet in Poland.

Single parrot sightings reported by ornithologists indicate that the problem of the occurrence of parrots in the wild in Poland is poorly understood. The aim of this study was to determine the species of parrots found in the wild, their numbers, the location of sightings and the time of year. We analysed the factors influencing the settlement of parrots in cities in other geographical locations [14,18,44]. We hypothesized that parrots would be more common in large cities and during the summer months and that the occurrence of parrots in the wild would be related to socio-economic factors, in particular the size of the human population. We hypothesized that higher financial status and education level of the population seem to increase the risk of parrots entering the natural environment. We paid special attention to the rose-ringed parakeet, as there are reports in the literature of breeding in this country. Our analyses aim to find out whether this breeding site was an isolated case and to what extent this invasive non-native species occurs in other locations in Poland.

## Material and methods

### Study area

Poland is the largest country in Central Europe with a population of more than 38 179 800 people. The largest city in the country is Warsaw (1.794 million inhabitants). Other large cities are Kraków (780,796 inhabitants), Łódź (664,071 inhabitants), Wrocław (642,700 inhabitants), Poznań (529,410 inhabitants), Gdańsk (628,592 inhabitants) and Szczecin (396,500 inhabitants). One of the largest population centers is the Silesian metropolitan area around Katowice (4.635 million inhabitants) [45]. Most of the country consists of lowland areas that are part of the European Plain. These merge in the south into highland areas and into the mountainous areas of the Carpathians and the Sudetes near the border with Ukraine, Slovakia and the Czech Republic. Most of the country lies in the basin of two rivers, the Vistula, which crosses the central part of the country, and the Oder, which forms the western border with Germany.

Poland lies in the temperate climate zone. In the higher parts of the Sudetes and the Carpathians, a mountain climate prevails. However, in the northwestern part of the country and in the lower reaches of the Oder river near the border with Germany, the climate has the characteristics of an oceanic climate with mild winters. The average annual temperature ranges from over 9°C near Wrocław, Legnica and Zielona Góra in the west of the country to about 6°C in the Suwałki region in the northeast. In summer, temperatures generally range from 18°C to 30°C, depending on the region. In winter, the highest temperatures are measured in the west of the country. They range from around 0°C in the Baltic coastal town of Świnoujście, -1°C in the Silesian Plain, Lubusz Land and the coast, -3°C in Warsaw to below -5°C in the north-east of the country near Suwałki, which is considered the Polish coldest area. On average, Poland has 3 to 5 heat waves and 2 to 4 cold waves per year. They comprise an average of 18 to 36 and 13 to 28 days per year, respectively. The number of days with snow cover varies and increases towards the east. In the Szczecin Lowland, the Lubusz Land and the Silesian Lowland the snow cover lasts less than 25 days per year, in the center of Poland about 60 days and in the north-east more than 100 days [46].

## Methods

The occurrence of parrots in the wild in Poland was recorded by analysing posts in the most popular Facebook group "Parrots lost and found" (in Polish: Papugi zaginione i znalezione) [47]. We collected data from October 2018, the whole of 2019 and from June to the end of December 2021 by analysing subsequent posts (a Facebook platform outage prevented us from collecting data from the beginning of 2021). We collected data on the parrot species (also to verify the attached photos), location of sighting, date, type of information: missing report, parrot captured by a person looking for the owner, sighting of a free flying parrot, and time spent in the wild. We analysed the records looking for information on whether the missing individual was found by the owner and whether the owner found the missing parrot. We noted whether the individual was alive or dead. Based on the reporter’s description of the sighting, we determined the type of environment in which the parrot was found. We distinguished between urban areas, forests and agricultural areas. Each time we analysed the date of capture and the descriptions of the reports to avoid describing the same individuals. For individuals where the species name was not given, e.g. rare and valuable parrots, we have determined the species from the attached photographs. In cases where the entry was vague but the genus was clearly stated, we considered the information to be that of the species most popular with breeders, e.g. rosella—eastern rosella *Platycercus eximius* (19 individuals), cockatoo—galah *Eolophus roseicapilla* (2 individuals), amazon—blue-fronted amazon *Amazona aestiva* (28 individuals). For all parrot species found in Poland, we use the term "non-native species" to describe their status [48].

We assigned the number of parrot individuals to 16 regions according to the administrative division of the country. The differences in parrot species diversity between regions were calculated using the Shannon-Wiener index. We correlated the number of parrot individuals in the regions with socio-economic data on the inhabitants of the region. The factors that we considered significant for the risk of parrots in the wild were the total number of inhabitants in the region, the number of people living in cities, the income of the inhabitants and their education, which we used as a proxy for the percentage of people with a university education. We also considered the number of older people > 65 years and younger people < 25 years [49] as factors that could influence the interest in amateur breeding of parrots and thus increase the risk of their escape. We also compared the relationship between the number of people living in a locality and the number of parrots captured in the wild. For this purpose, we used statistical data on the number of inhabitants of localities available on websites [50,51]. Based on the available data, we also classified the status of the locality and described it as rural or urban, regardless of the number of inhabitants. We analysed the escapes of the rose-ringed parakeet, a species breeding in Poland, paying attention to the number of individuals present in a locality.

### Statistical analysis

A total of 2,567 reports of parrots observed in the wild were analysed (S1 Appendix). We calculated the percentage of individuals of each species in relation to the total number of parrots. The frequency of occurrence of all parrot species and the rose-ringed parakeet in consecutive months of the year was tested using the chi-square test for goodness of fit with a uniform distribution. The strength of the relationship between the total number of occurrences and demographic data aggregated to provinces was analysed using Pearson’s linear correlation significance test. We analysed the relationship between the human population at 781 locations and the number of reported parrot sightings using linear regression. The method of minimizing the mean square error was used to estimate the parameters of the linear model. Its adequacy was tested using the F-Snedecor test. For all statistical tests, we used the significance level *p* = 0.05. All analyses were performed with R [52].

## Results

### Parrots found in the wild in Poland

During the period from autumn 2018 to the end of 2019 and from June to the end of 2021, there were 2,567 reports of parrots escaped, found or observed in the wild. A single report involved an average of 1.04 individuals (range: 1–6). In total, information was collected on 2,675 individuals of parrots belonging to 49 species. The most common species were cockatiels—962 individuals, budgerigars—884 individuals and rose-ringed parakeet—182 individuals (Table 1). We analysed information on 43 cases where the length of stay of the parrot in the wild was known, and it averaged 5.6 days. (range: 1–90 days).

**Table 1:** The number of observations per species for each parrot species reported in the wild in Poland.

Species	N	%
Cockatiel *Nymphicus hollandicus*	962	35.96
Budgerigar *Melopsittacus undulatus*	884	33.05
Rose-ringed parakeet *Psittacula krameri*	182	6.80
Rosy-faced lovebird *Agapornis roseicollis*	105	3.93
Eastern rosella *Platycercus eximius*	92	3.44
Monk parakeet *Myiopsitta monachus*	45	1.68
Red-rumped parrot *Psephotus haematonotus*	43	1.61
Crimson-bellied parakeet *Pyrrhura perlata*	37	1.38
Crimson rosella *Platycercus elegans*	35	1.31
Grey parrot *Psittacus erithacus*	34	1.27
Turquoise-fronted amazon *Amazona aestiva*	33	1.23
Red-crowned parakeet *Cyanoramphus novaezelandiae*	25	0.93
Alexandrine parakeet *Psittacula eupatria*	24	0.90
Blue-and-yellow macaw *Ara ararauna*	23	0.86
Superb parrot *Polytelis swainsonii*	20	0.75
Derbyan parakeet *Psittacula derbiana*	16	0.60
Australian ringneck *Barnardius zonarius*	11	0.41
Sun parakeet *Aratinga solstitialis*	9	0.34
Regent parrot *Polytelis anthopeplus*	9	0.34
Jandaya parakeet *Aratinga jandaya*	7	0.26
Green-cheeked parakeet *Pyrrhura molinae*	7	0.26
Blue-winged macaw *Primolius maracana*	6	0.22
Galah *Eolophus roseicapilla*	6	0.22
Eclectus parrot *Eclectus roratus*	6	0.22
Red-breasted parakeet *Psittacula alexandri*	5	0.19
Yellow-headed amazon *Amazona oratrix*	5	0.19
Senegal parrot *Poicephalus senegalus*	5	0.19
Plum-headed parakeet *Psittacula cyanocephala*	4	0.15
Red-winged parrot *Aprosmictus erythropterus*	3	0.11
Coconut lorikeet *Trichoglossus haematodus*	3	0.11
Australian king parrot *Alisterus scapularis*	3	0.11
Festive amazon *Amazona festiva*	2	0.07
Black-headed parrot *Pionites melanocephalus*	2	0.07
Mitred parakeet *Psittacara mitratus*	2	0.07
Burrowing parrot *Cyanoliseus patagonus*	2	0.07
Pale-headed rosella *Platycercus adscitus*	2	0.07
Northern rosella *Platycercus venustus*	2	0.07
Barred parakeet *Bolborhynchus lineola*	2	0.07
Pacific parrotlet *Forpus coelestis*	2	0.07
Orange-winged amazon *Amazona amazonica*	1	0.04
Red-and-green macaw *Ara chloropterus*	1	0.04
Scarlet macaw *Ara macao*	1	0.04
Golden-collared macaw *Primolius auricollis*	1	0.04
Green-thighed parrot *Pionites leucogaster*	1	0.04
Blue-crowned parakeet *Thectocercus acuticaudatus*	1	0.04
Princess parrot *Polytelis alexandrae*	1	0.04
Rainbow lorikeet *Trichoglossus moluccanus*	1	0.04
Yellow-collared lovebird *Agapornis personatus*	1	0.04
Sulphur-winged parakeet *Pyrrhura hoffmanni*	1	0.04
**Total (49 species)**	**2,675**	**100**

Most of the data on parrots (79.77%) referred to four species that are not subject to trade restrictions related to CITES and EU law: cockatiel, budgerigar, rose-ringed parakeet and rosy-faced lovebird. We collected information on sightings of 541 individuals (20.22%) belonging to the group subject to compulsory registration according to CITES and the corresponding EU regulations.

### Seasonal determinants of the appearance of parrots in the wild in Poland

**Fig 1:**
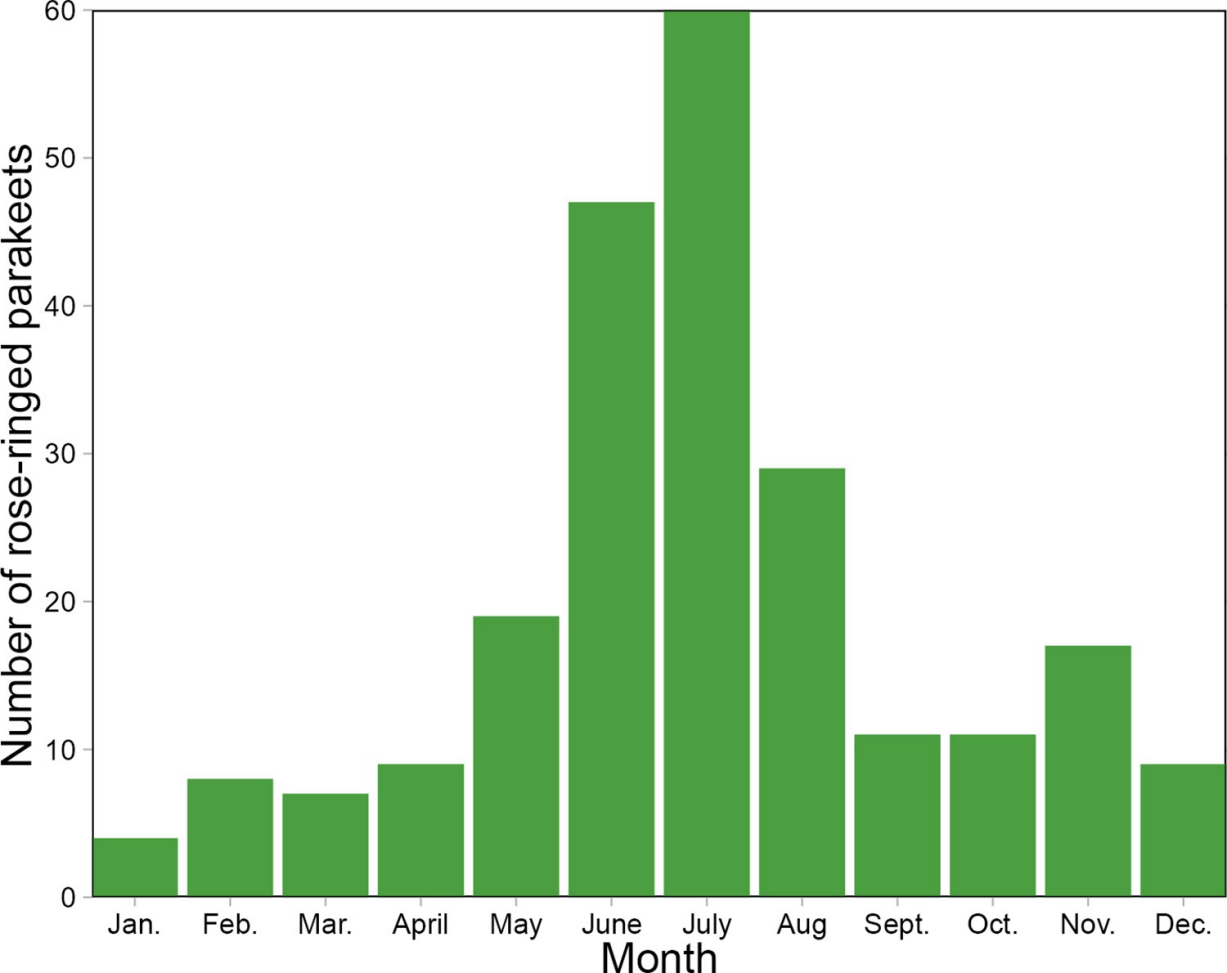
Number of parrots observed in Poland in each month.

Parrots were found in the wild all year round, even in the winter months. There were significant differences between the number of parrots in each month (χ^2^ = 2678.8, df = 11, p < 0.001). The highest number of parrots in the wild was recorded in summer, in June and July (50%), and the lowest in winter from December to March (7%) (Fig 1).

### Lost or found? How often do parrots observed at large return to their owners?

Information about lost parrots (47.21%) was similar in number to information about found parrots (41.12%). The third category was information about parrot sightings, which accounted for 18.95% of the data. Of the 1,100 individuals found, the majority of parrots were alive (99.09%). Only 10 dead individuals were found (0.91%), including five budgerigars, three cockatiels and one each of a red-crowned parakeet *Cyanoramphus novaezelandiae* and an Australian ringneck *Barnardius zonarius*. Dead individuals were found from May to July, only one individual was found in December during the winter period. The situation where the same individual was reported missing and then found by its owner applied to 7.28% of the animals.

Most parrots (N = 2669) were found in urban areas (99.88%), of which 80.59% were observed in cities and 19.40% in villages. Twice parrots were observed in agricultural areas and once they were found in forests. Parrots in the wild were recorded in 786 localities. In 96 localities (92 cities, four villages), five or more individuals per locality were found, which corresponds to 64% of all individuals recorded. Most parrots in the wild were found in large cities, led by the capital Warsaw with 220 individuals, Poznań with 121 individuals, Kraków with 86 individuals, Wrocław with 82 individuals, Łódź with 67 individuals, Gdańsk with 64 individuals, Szczecin with 45 individuals and Katowice with 40 individuals.

### Observations of rose-ringed parakeet in the wild in Poland

**Fig 2:**
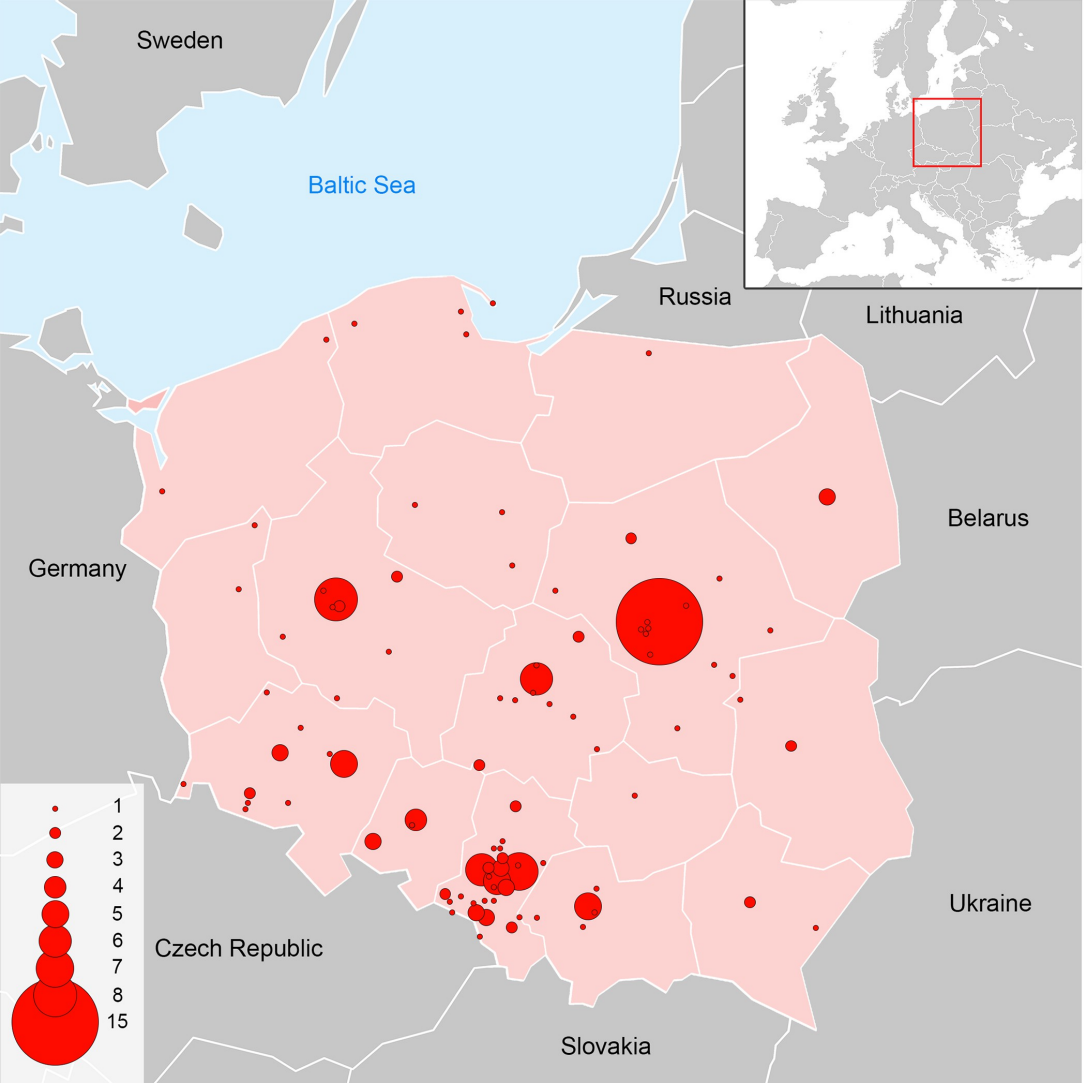
Number of rose-ringed parakeet *Psittacula krameri* observed in Poland. The size of the circles corresponds to the number of parrots observed at localities. The maps was redrawn on the basis of https://www.cia.gov/the-world-factbook/static/cd0ba07f4edc52b9f8e10b9992267c52/europe_pol-1.pdf and https://stat.gov.pl/en/regional-statistics/classification-of-territorial-units/administrative-division-of-poland/.

We collected information on 182 individuals of rose-ringed parakeet. On average, 1.82 individuals were found per location (range: 1–15 individuals). Most of the information (84.06%) came from cities and 15.93% from villages. Rose-ringed parakeets were found at 100 localities, of which 38.46% were single observations. Between two and 15 specimens of rose-ringed parakeets were observed at 30 sites. The highest number of rose-ringed parakeets was found in Warsaw (15 individuals), Poznań (8 individuals), Sosnowiec (7 individuals), Gliwice and Łódź (6 individuals each), Kraków, Ruda Śląska and Wrocław (5 individuals each) (Fig 2).

Information on individuals that escaped from amateur breeding predominates (59.34%). Individuals seen in the wild and for which information was published by persons other than the owners accounted for 20.32% of the total number of rose-ringed parakeets. 29.12% of these parrots were captured, and 15.74% of the data set was accounted for by individuals known to have been returned to their owners after escape.

**Fig 3:**
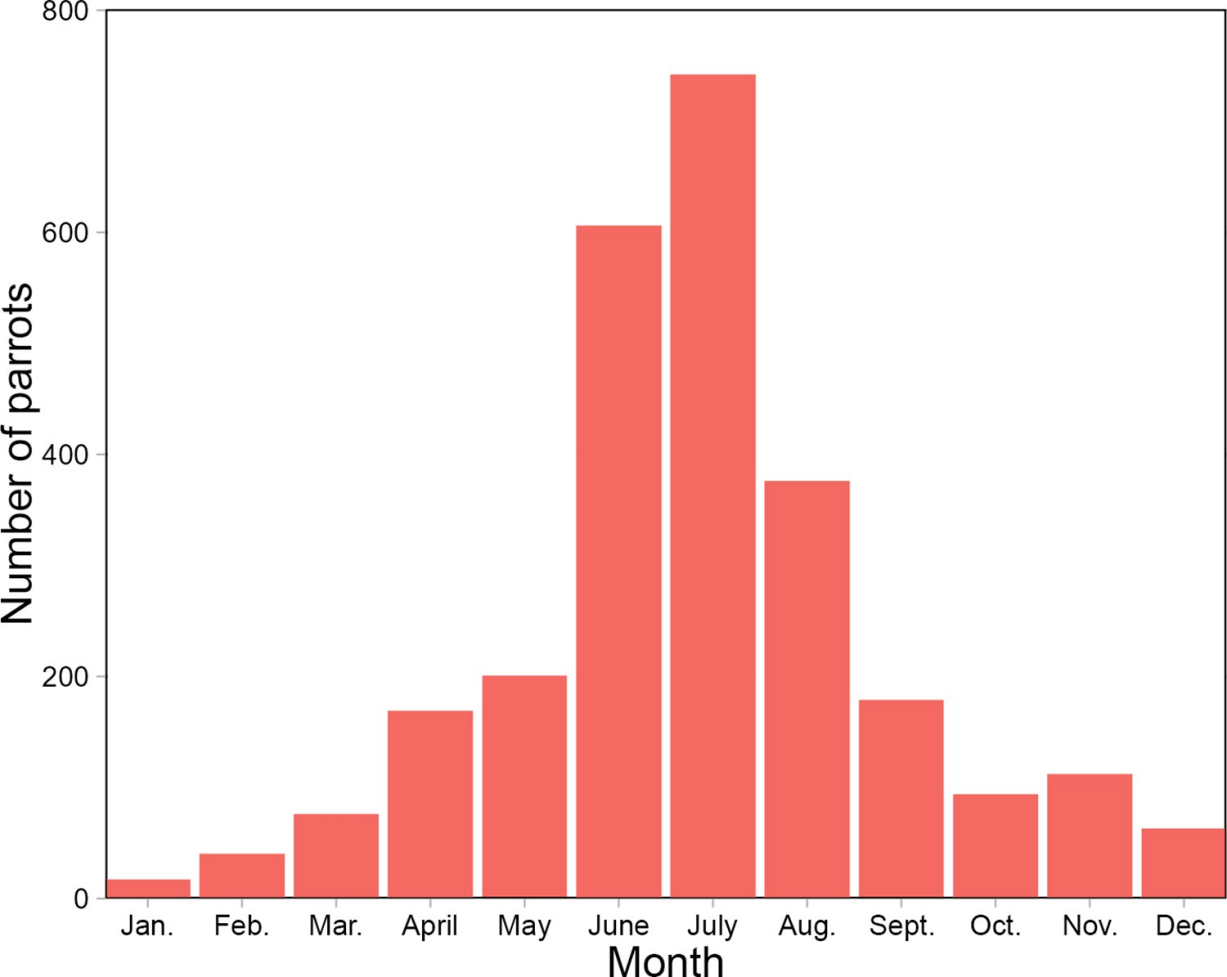
Number of rose-ringed parakeet *Psittacula krameri* observed in Poland in each month.

There were significant differences in the number of rose-ringed parakeets between months (χ^2^ = 175.91, df = 11, p < 0.001). Rose-ringed parakeets are most commonly observed in the summer months. However, they can be found throughout the year. In the coldest months of the year, from November to the end of March, 21.85% of all rose-ringed parakeets were found (Fig 3).

### Socio-economic factors influencing the emergence of parrots in the wild

**Fig 4:**
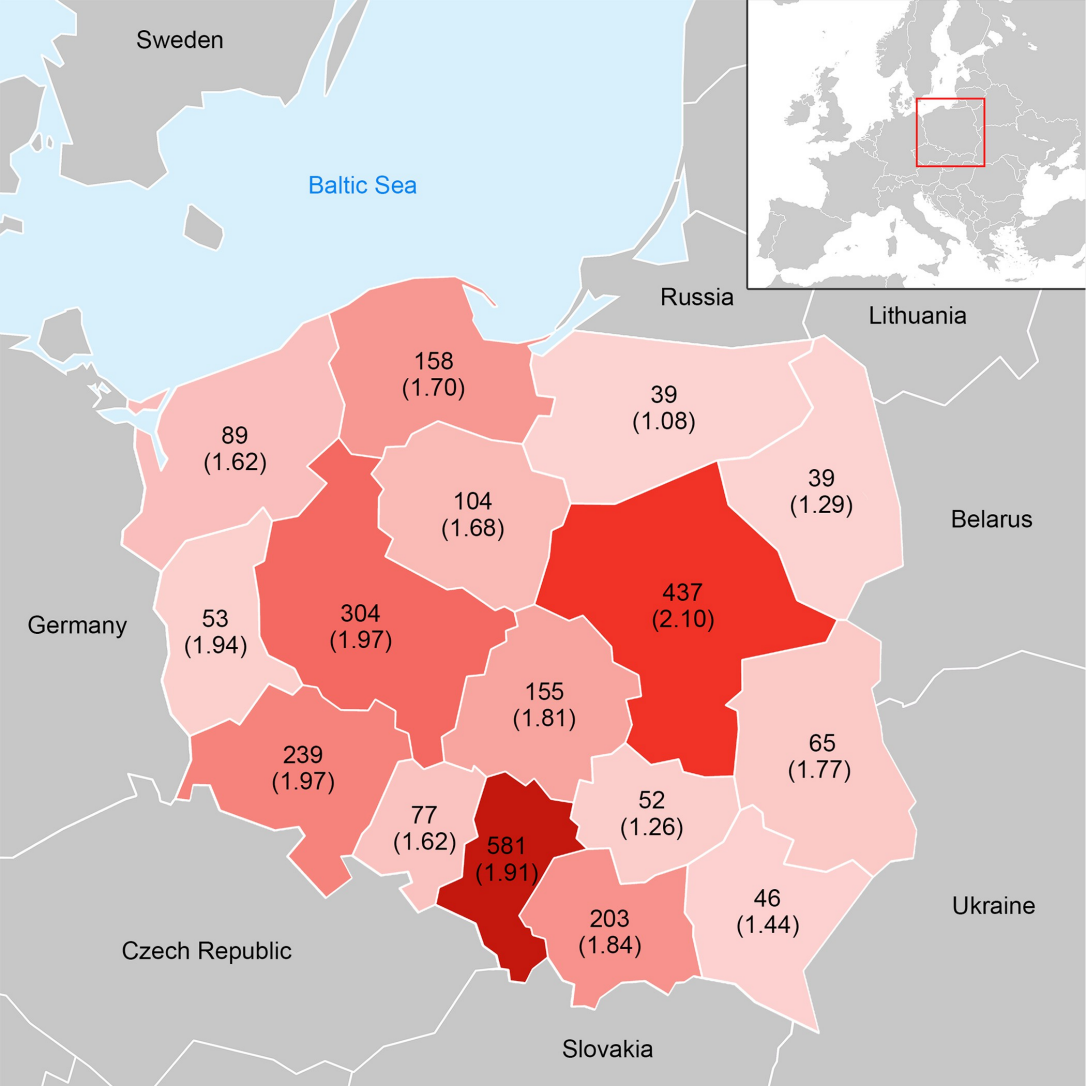
Number of parrots found in the administrative regions of Poland. Areas with higher parrot occurrence are marked with darker red color. The Shannon-Wiener index of parrot species diversity in the regions is given in brackets. The maps was redrawn on the basis of https://www.cia.gov/the-world-factbook/static/cd0ba07f4edc52b9f8e10b9992267c52/europe_pol-1.pdf and https://stat.gov.pl/en/regional-statistics/classification-of-territorial-units/administrative-division-of-poland/.

**Fig 5:**
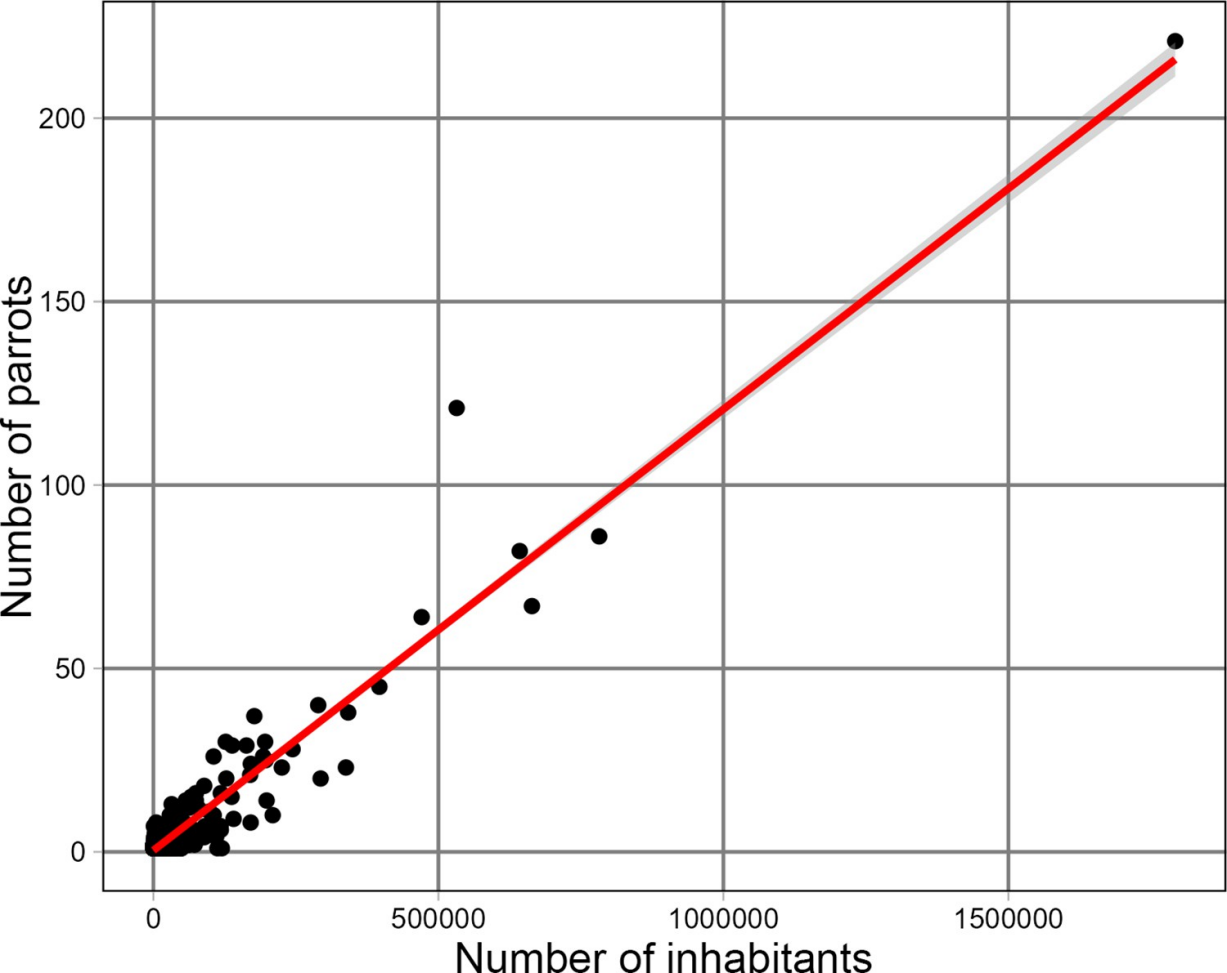
Influence of the number of inhabitants of the locality where parrots were found on the number of parrots observed in the wild.

One socio-economic factor that influences the number of reported parrot sightings or escapes in regions is the size of the resident population. The parameter estimate of the linear model shows that on average 1.2 parrots per 10,000 inhabitants occur in the wild in Poland. Parrots are reported more frequently in regions with a greater human population (Fig 4) and a higher gross income of the population. The number of parrots in the wild is also positively correlated with the proportion of the population that has a university degree (Table 2). A comparison of the number of inhabitants in the urbanized areas where parrots were observed with the number of parrots found in the wild shows that as the number of inhabitants increases, the number of parrots found in the wild also increases (F _1, 779_ = 8063, p < 0.001) (Fig 5). Species diversity values for the regions based on the standard Shannon-Wiener index show that the highest species diversity was found in the region with capital city and the lowest in the northeastern region (Fig 4).

**Table 2:** Socio-economic factors influencing the number of parrots in the wild in Poland. Bold p-values are statistically significant (p < 0.05).

Variable	Estimate	p-value
total population of the region	0.909	**0.0000**
average gross income	0.729	**0.0013**
percentage of people with university degree	0.543	**0.0295**
% of population in urban areas	0.534	**0.0329**
% of population over 65 years of age	0.131	0.6278
% of population under 25 years of age	0.038	0.8883

## Discussion

This is the first study to describe how many parrot specimens escape from amateur breeding in Poland into the wild. The results of our study support the hypothesis that parrots in the wild are most common in large cities and in regions of higher socio-economic status. For many years, it was believed that the political isolation of Central Europe during the Cold War and restrictions on international trade prevented the introduction of non-native bird species [53]. The results of our study indicate that the problem of the appearance of such popular non-native pet species as parrots affects the entire territory of Poland and especially large cities. We are currently witnessing the appearance of the first breeding site of the rose-ringed parakeet in Nysa [43]. Our results show that many individuals of this species are appearing in the wild in many other cities in the country, which could lead to the emergence of new breeding sites.

Parrots are found in the European fauna mainly in the western and southern parts of the continent. The eastern limit of the parrots’ range is defined at the German-Polish border [16]. Recent studies do not list Poland among the countries where parrots have naturalized and established breeding populations [15,16,22]. The results of our study suggest that parrots can be observed in Poland in urban areas throughout the year and the number of individuals per year can be in the thousands. The main reasons for the emergence of parrots in the wild are their escapes from breeding and deliberate releases. In addition, the number of naturalized parrots correlates with the number of escapes from amateur breeding [14,15]. The most important factor determining the abundance of non-native bird species is human-induced propagule pressure, which is expressed in the number of introduced species. The start of the invasion process is often poorly understood due to a lack of data [53]. However, studies from New Zealand show that the probability of a male/female pair being present among individuals escaping from captivity in a single location is over 80% [14].

Most parrot species are traded both internationally and domestically, and are transported between often distant regions of the world. It is difficult to estimate the number of captive-bred individuals of the four parrot species most commonly found in the wild in Poland—cockatiel, budgerigar, rose-ringed parakeet and rosy-faced lovebird. Cockatiel, budgerigar and rose-ringed parakeet were the most common species found in the wild in surveys based on social media analysis [14]. Their availability in the trade is high, which is also associated with a low price. Other parrot species are subject to trade restrictions, e.g. CITES, and registration requirements for live birds. Trade of common, least threatened IUCN-status parrot species is increasing worldwide [2]. Preliminary analyses of the CITES parrot species registered in Polish government institutions indicate that the mandatory registration of animals is not being implemented to a sufficient extent (Ważna, unpublished material).

The scale of the emergence of parrots in Poland is many times greater than the data published in the national ornithological literature would suggest. Individual sightings of rose-ringed parakeet, Derbyan parakeet, crimson rosella, eastern rosella, monk parakeet, yellow-collared lovebird by ornithologists are published in the annual activity reports of the Faunistic Commission, in which observations of rare and unusual species are reported [31,42]. Such sightings of parrots in the wild have status E—unnatural occurrence. No parrots in the wild were reported in 2020 in Poland [54,55].

The vast majority of parrot sightings in Poland take place in urban areas. Parrots in Europe (and also in other parts of the world) prefer large urban metropolitan areas in the colder months due to the urban heat island effect [14]. Cities also provide shelter in the form of thermal insulation of buildings, which offers better protection than natural hollows [16]. During winter, survival is facilitated by the widespread provision of bird food through bird feeders [56]. In urban areas in temperate climates, parrots use bird feeders all year round [57]. Harsh climatic conditions are an additional factor that encourages parrots in colder regions to establish populations in urban areas where they are most likely to survive [58].

The cockatiel is the most abundant species in the wild in Poland. It forms a breeding population in western Europe, which occasionally also includes Germany [16]. Originally from Australia, it requires high ambient temperatures, which are higher than those occurring in Polish cities in winter. The second most common species in Poland—the budgerigar—is similarly sensitive to cold. The most common parrot species observed in US cities is the monk parakeet, which makes up only a small percentage of the parrots found in Poland. However, this species is able to establish breeding populations in cities with cold climates such as Chicago or New York [17,59–61]. In Europe, the monk parakeet is known to breed in colder parts of the continent, e.g. in Denmark [62].

**Fig 6:**
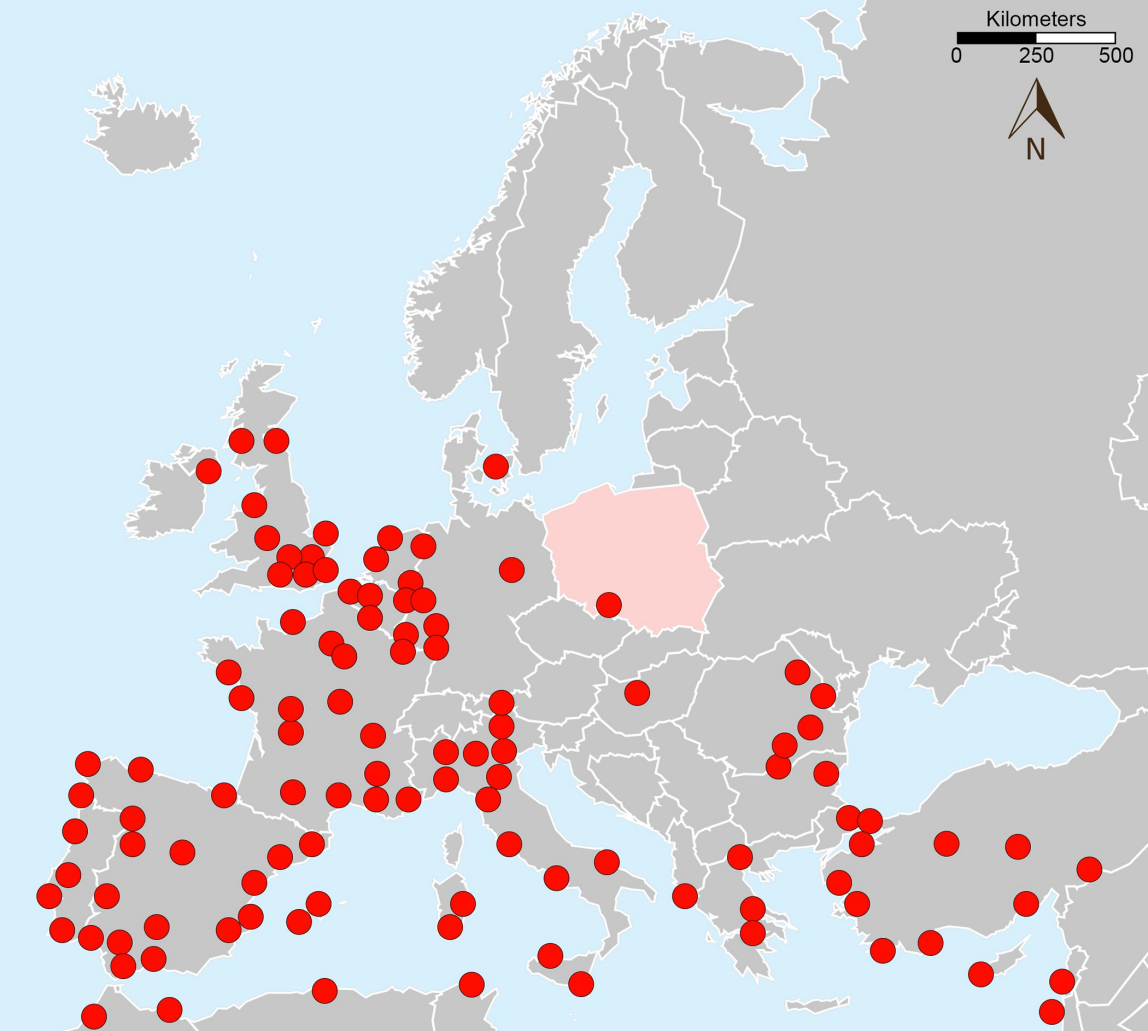
European distribution of the rose-ringed parakeet *Psittacula kramerii* [16,43]. The maps was redrawn on the basis of https://www.cia.gov/the-world-factbook/static/cd0ba07f4edc52b9f8e10b9992267c52/europe_pol-1.pdf.

The species with the highest probability of long-term residence in the environment is the rose-ringed parakeet, the third most numerous species in our study. It currently forms breeding populations in 10 European countries, and their numbers are steadily increasing [23] (Fig 6). The status of the species is unknown in Ireland, Switzerland and Ukraine, where there is data on observations of the species but no breeding sites [22]. The breeding pairs of rose-ringed parakeet closest to the Polish border were observed in Berlin for several years in the 1990s [63]. After that, however, no more breeding pairs were observed [64]. In Poland, the parakeet clutch found in the southwestern part of the country indicates that the species probably winters there. After the first clutch of a rose-ringed parakeet was found in Poland in 2018, no further clutches of this species were observed at the only Polish nesting site in the following years. However, the results of our study suggest that the unclear status of the rose-ringed parakeet’s only native nesting site may also be temporary. However this cavity-nesting species is cryptic and some broods may remain undetected. While we were collecting data for this study, we found posts on social media documenting the next cases in which three specimens of this parrot had escaped from breeding into the wild in Nysa within a short period of time. We also found about 40 individuals in our study that were observed during the autumn and winter months. In western Poland, where the climate is mild in the lowlands, it is likely that rose-ringed parakeet is wintering, especially in urban areas.

The level of development of the Polish administrative area varies. The western part is home to most of the large cities and is more industrialized, which is reflected in the higher income of the inhabitants. In the eastern part, outside the Warsaw metropolitan area (the country’s capital), rural areas predominate with a characteristic fragmentation of farms. These factors have a significant impact on the diversity and conservation of native bird species [65]. The western and eastern parts of the country also differ significantly in climatic conditions. The western lowland regions have a mild climate with maritime climatic influences and a long growing season. The eastern part has a typical continental climate with cold and mostly snowy winters. The differences in land use and environmental conditions suggest that the risk of parrots settling and establishing a breeding population in the eastern part of the country is low. The western part of Poland, especially around cities such as Szczecin, Poznań and Wrocław, seems to fulfill the conditions for at least temporary survival of parrots in the wild, especially considering the observed extent of parrot presence in the wild. The example of the breeding population in Nysa [43] shows that this also applies to small towns where individuals may also be present. An analysis of social media posts suggests that in many cases where parrots appear in the wild, deliberate release is the reason. In some cases, the owner releases the birds in the belief that they will return to him. Owners do not comment on cases where they deliberately get rid of their pets for obvious reasons, which is extremely easy in the case of birds.

Socio-economic factors influencing the development of urbanized areas have a significant impact on the conservation status of native fauna [66,67]. The correlation between the occurrence of the barn owl *Tyto alba* and the number of people employed in agriculture in Poland (where the educational level of workers is low) has a very different dimension in the case of parrots, and it is likely that this also applies to other non-native species [65]. The educational level of the population and the associated financial status seem to increase the risk of non-native species bred as pets entering the natural environment. It is suggested by the correlation found in other studies between an increased trade in parrots and a higher per capita GDP [2]. Human population size is most strongly correlated with the diversity of non-native species [68]. The number of parrots in the wild in Polish cities is correlated with the population size of the inhabitants. This relationship has also been confirmed in other studies [14]. In our study, we could not confirm the correlation between the number of parrots recorded and the proportion of elderly people in the population. A correlation between the abundance of parrots and the age of the population has been described in Spain, where parrots were observed in areas where older people resided, who were presumably more willing to feed the parrots [18]. In Poland, however, parrots were not popular pets for a long time and it was only the rise in the material status of families after Poland joined the European Union that led to an increase in trade in these birds. Nevertheless, the higher salaries apply mainly to those who work and live in large cities. However, a high level of education among the population is not enough to prevent the release of non-native species into the natural environment. Experts consider that the wildlife trade is sustained mainly by young consumers from higher socio-economic groups and educational backgrounds [69].

The impact of non-native parrot species on the natural ecosystem is not fully understood [70]. There are no reports on this issue in Poland. Certainly, parrots can transmit many diseases, but the importance of parrots as vectors of pathogens for native species also needs further research [71,72]. Non-native species generally pose a threat to native wildlife and can cause severe environmental damage [73–79]. The problem is also poorly recognized because parrots of the same species can inhabit a variety of habitats in Europe [28]. Little information is currently available on the rose-ringed parakeet’s interactions with native fauna. Its presence in the habitat can probably affect the foraging behavior of native bird species, among others [80]. Cases of this species attacking cavity-dwelling bats have also been reported in Italy and Spain [74,81,82].

Knowledge about the population of non-native parrots is not complete. Using data from social media and other online sources, it is possible to collect high quality data on the occurrence, biology and dispersal of parrot species [22,58,83]. This fits into a broader scientific trend in species ecology known as iEcology [84]. Analysis of the data available on the iBirds and iNaturalst platforms, collected as part of so-called citizen science, will allow various behaviours such as feeding, nesting and roosting in other species to be studied to better understand acclimation to high latitude regions.

The likelihood of a successful invasion of an non-native species depends on a number of factors, the most important of which are climate and environmental conditions [58]. It was previously thought that the temperate climate of central Europe had a negative impact on the invasion success of the rose-ringed parakeet, the most widespread parrot species in Europe [23]. Our studies on the occurrence of parrots in the wild in Poland show that the hundreds of individuals found especially in large cities within a short period of time are not just incidental cases of these birds escaping from amateur breeding. Given the increasingly rare cold winters and global warming, we can assume that the increase in occurrence of non-native parrot nesting sites in Polish cities is only a matter of time. The probability that these populations will die out in harsh winters is high. However, the number of individuals introduced through amateur breeding indicates that parrots could become an increasingly common element of the fauna of Polish cities.

## Supporting information

S1 Appendix Number of parrots found in the wild in Poland. (XLSX)
